# Correction: Effect of a loss of the *mda5/ifih1* gene on the antiviral resistance in a Chinook salmon *Oncorhynchus tshawytscha* cell line

**DOI:** 10.1371/journal.pone.0338234

**Published:** 2025-12-04

**Authors:** Catherine Collins, Lise Chaumont, Mathilde Peruzzi, Nedim Jamak, Pierre Boudinot, Julia Béjar, Patricia Moreno, Daniel Álvarez Torres, Bertrand Collet

The Funding statement for this article is incorrect. The correct Funding statement is as follows: This project was funded in part by the European Union through AQUAEXCEL3.0 (Grant Agreement 871108) and AQUAFAANG (Grant Agreement 817923), by the Research Council of Norway through the project PMCV (Project 301083) and by the Spanish Government through the project PID2020-115954RB-I00. LC was a recipient of PhD funded by Virbac and the French Association for Research and Technology (ANRT) [Convention CIFRE #2020/0646] in collaboration with the Fish Infection and Immunity laboratory (INRAE, VIM, Jouy-en-Josas, France). The funders had no role in study design, data collection and analysis, decision to publish, or preparation of the manuscript.

## References

[1] Collins C, Chaumont L, Peruzzi M, Jamak N, Boudinot P, Béjar J, et al. Effect of a loss of the *mda5/ifih1* gene on the antiviral resistance in a Chinook salmon *Oncorhynchus tshawytscha* cell line. PLoS One. 2024;19(10):e0311283. doi: 10.1371/journal.pone.0311283 39401233 PMC11472919

