# Supplementary figures and images for: Effect of a loss of the mda5/ifih1 gene on the antiviral resistance in a Chinook salmon Oncorhynchus tshawytscha cell line
Source: PLoS One. 2024 Oct 14;19(10):e0311283. doi: 10.1371/journal.pone.0311283 (PMC11472919; doi:10.1371/journal.pone.0311283)

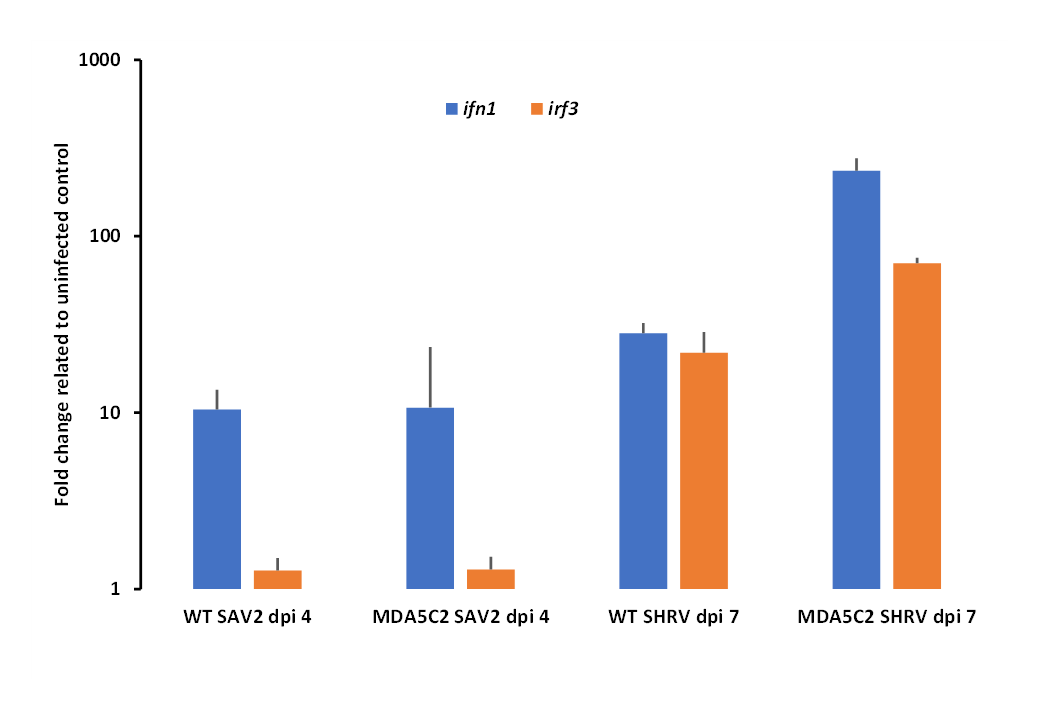

Supplement: S1 Fig — Data represent the average fold change (N = 3) in the transcription levels of ifn1 or irf3 + standard deviation. (TIF) [file pone.0311283.s001.tif]
