# Supplementary material for: Effect of a loss of the mda5/ifih1 gene on the antiviral resistance in a Chinook salmon Oncorhynchus tshawytscha cell line
Source: PLoS One. 2024 Oct 14;19(10):e0311283. doi: 10.1371/journal.pone.0311283 (PMC11472919; doi:10.1371/journal.pone.0311283)
Supplement: Attachment — Submitted filename: Response to Reviewers.docx [file pone.0311283.s003.docx]

Journal Requirements:

1. When submitting your revision, we need you to address these additional requirements.

Please ensure that your manuscript meets PLOS ONE's style requirements, including those for file naming. The PLOS ONE style templates can be found at 
<https://journals.plos.org/plosone/s/file?id=wjVg/PLOSOne_formatting_sample_main_body.pdf> and <https://journals.plos.org/plosone/s/file?id=ba62/PLOSOne_formatting_sample_title_authors_affiliations.pdf>

*The manuscript has been reformatted to meet PLOS ONE’s style. Figures files have been uploaded (TIF format) as well as a supporting file and supplementary figure.*

2. We suggest you thoroughly copyedit your manuscript for language usage, spelling, and grammar. If you do not know anyone who can help you do this, you may wish to consider employing a professional scientific editing service.

The American Journal Experts (AJE) (<https://www.aje.com/>) is one such service that has extensive experience helping authors meet PLOS guidelines and can provide language editing, translation, manuscript formatting, and figure formatting to ensure your manuscript meets our submission guidelines. Please note that having the manuscript copyedited by AJE or any other editing services does not guarantee selection for peer review or acceptance for publication.

Upon resubmission, please provide the following:

The name of the colleague or the details of the professional service that edited your manuscript

A copy of your manuscript showing your changes by either highlighting them or using track changes (uploaded as a *supporting information* file)

A clean copy of the edited manuscript (uploaded as the new *manuscript* file)”

*The first author is a native English-speaking scientist with a long-standing experience in publishing in peer-reviewed scientific journal both as an author and as an editor. Both reviewers have replied “yes” to the question 4. The first author has been through the whole manuscript before submission. Nevertheless, the revised version has been double checked and some of the sentences were improved. We believe that the level of English is perfectly acceptable.*

3. We note that the grant information you provided in the ‘Funding Information’ and ‘Financial Disclosure’ sections do not match.

When you resubmit, please ensure that you provide the correct grant numbers for the awards you received for your study in the ‘Funding Information’ section.

*Grant number and correct funding Information have been verified and completed on the online submission system.*

4. Thank you for stating the following in the Acknowledgments Section of your manuscript: "We are grateful to Stéphane Biacchesi and Emilie Mérour (INRAE, Jouy-en-Josas, France) for providing the SAV inoculum and to Pr. Niels Lorenzen (National Institute for Aquatic Resources, Technical University of Denmark, Denmark) for providing the SHRV isolate. This project was funded in part by the European Union through AQUAEXCEL3.0 (Grant Agreement 871108) and AQUAFAANG (Grant Agreement 817923) and by the Research Council of Norway through the project PMCV (Project 301083). LC was a recipient of PhD funded by Virbac and the French Association for Research and Technology (ANRT) [Convention CIFRE #2020/0646] in collaboration with the Fish Infection and Immunity laboratory (INRAE, VIM, Jouy-en387 Josas, France).

We note that you have provided funding information that is not currently declared in your Funding Statement. However, funding information should not appear in the Acknowledgments section or other areas of your manuscript. We will only publish funding information present in the Funding Statement section of the online submission form.

Please remove any funding-related text from the manuscript and let us know how you would like to update your Funding Statement. Currently, your Funding Statement reads as follows: "The funders had no role in study design, data collection and analysis, decision to publish, or preparation of the manuscript."

Please include your amended statements within your cover letter; we will change the online submission form on your behalf.

*This has been addressed in the revised version.*

5. We note that your Data Availability Statement is currently as follows: "All relevant data are within the manuscript and its Supporting Information files."

Please confirm at this time whether or not your submission contains all raw data required to replicate the results of your study. Authors must share the “minimal data set” for their submission. PLOS defines the minimal data set to consist of the data required to replicate all study findings reported in the article, as well as related metadata and methods (<https://journals.plos.org/plosone/s/data-availability#loc-minimal-data-set-definition>).

For example, authors should submit the following data:

- The values behind the means, standard deviations and other measures reported;
- The values used to build graphs;
- The points extracted from images for analysis.

Authors do not need to submit their entire data set if only a portion of the data was used in the reported study.

If your submission does not contain these data, please either upload them as Supporting Information files or deposit them to a stable, public repository and provide us with the relevant URLs, DOIs, or accession numbers. For a list of recommended repositories, please see <https://journals.plos.org/plosone/s/recommended-repositories>.

If there are ethical or legal restrictions on sharing a de-identified data set, please explain them in detail (e.g., data contain potentially sensitive information, data are owned by a third-party organization, etc.) and who has imposed them (e.g., an ethics committee). Please also provide contact information for a data access committee, ethics committee, or other institutional body to which data requests may be sent. If data are owned by a third party, please indicate how others may request data access.

*We added a supporting Information file (excel) containing the individual data that were used to establish the figures 3, 4 and 5 (qPCR experiments).*

5. We note that you have included the phrase “data not shown” in your manuscript. Unfortunately, this does not meet our data sharing requirements. PLOS does not permit references to inaccessible data. We require that authors provide all relevant data within the paper, Supporting Information files, or in an acceptable, public repository. Please add a citation to support this phrase or upload the data that corresponds with these findings to a stable repository (such as Figshare or Dryad) and provide and URLs, DOIs, or accession numbers that may be used to access these data. Or, if the data are not a core part of the research being presented in your study, we ask that you remove the phrase that refers to these data.

*Some statement “data not shown” corresponded to intermediate analyses i.e. sequencing of the polyclonal cell population after transfection with sgRNAs which is not necessary for the replication of the work – they have therefore been deleted in the revised version.*

Additional Editor Comments:

The manuscript is of interest for the PLOS ONE readers, however, the authors should correct and resolve all the questions raised by the reviewers, in order to be accepted for publication.
**Reviewers' comments:
Reviewer #1:**

The present manuscript explores the role of Melanoma Differentiation-Associated protein 5 (MDA5) in antiviral defense in Chinook salmon cells, using CRISPR/Cas9 to create a cell line lacking the ifih1 gene. The study found that while the induction of type I interferon (IFN1) was impaired in these modified cells upon viral infection, there was no increase in cytopathic effects. These results suggest that the cells have redundant antiviral defense mechanisms that compensate for the loss of MDA5.

Undoubtedly, a lot of work has gone into this paper and it is characterized by novelty. However, some questions arise.

Could the authors explain an alternative mechanism path?

*It is difficult to propose an alternative pathway without being speculative. This pathway would allow the cells to retain viral resistance in the absence of type I IFN induction. This may be related to viral subversion where, in wild type cells, although ISGs are induced their activity may be blocked by some specific viral proteins. This could explain that we cannot see any differences in the viral cytopathic effect between our ifih1^-/-^ and wt cells.*

Given that RIG-I and MDA5 target different dsRNA lengths, could this variability be affecting the discussion?

*This statement refers to knowledge from mammalian model literature and does not necessarily apply to fish model and this has not been investigated fully in fish. Again, it is difficult to answer without being speculative considering our work focus on mda5. Comparative analysis of rigI^-/-^ and ifih1^-/-^ is required to answer this question.*

In lines 70-81, the text is overly verbose. Since other species also possess the specific gene, I strongly recommend moving this section to the supplementary material. The authors could also include other fish species (e.g.see genus: Gadus, Perka) for a comparative sequencing analysis.

*We would like to keep this section as it is. It is designed to give an update on the* ***published*** *literature on fish mda5. We are aware that the number of species is large but we do think this important to illustrate the characteristics of the fish immunology scientific community: i) it is fragmented by the large diversity of species studied ii) there is a lack of functional tools since despite the effort that have been spent on the study of this particular gene there is no significant progress on the identification of its function in the fish model.*

In the manuscript, the authors state "data not shown" three times. They should either provide this data in the supplementary material or omit these repeatead references to refine the text.

*We have removed one of the “data not shown” related to the sequencing verification of the polyclonal edited population which is an intermediate stage of the method. Other data not shown statement have been replace by reference to previous published work in one instance and provision of a supplementary figure in the other instance.*

Are there any available results from at least the wild type analysis? Also, the position 20 is not significant on the structure for protein functionality.

*We have added a figure in the supplementary file showing that in both WT and MDA5C2 cell lines the IFN1 pathway is fully functional.*

Although the maximum likelihood method is accurate for this type of phylogenetic analysis, the authors should discuss the results more thoroughly and could consider using another model for the matrix.

*This work is not a phylogenetic analysis of fish mda5 – this has been done extensively by others. The purpose of the tree in Fig 1 is to ensure that the chinook salmon LOC112225192 gene was indeed the orthologue of mammalian ifih1 gene.*

Based on these facts, I suggest the conditional acceptance of this manuscript for publication in PlosOne.

**Reviewer #2:**

The present report explores knockdown of a key receptor in PAMP recognition, contributing to antiviral immunity. Knockdown MDA5 cells were created using CRISPR/Cas9 and cells were infected with several important fish pathogens to look at differences in responses. Feedback is given in point form below.

- In all figures the knockdown cells are not compared to wildtype cells in figures. It was comment upon in the results that there were no differences between MDA5C2 and WT cells (225). This is misleading to then continue describing only the knockdown clones. This data must be shown in all figures.

*We have shown that there was no impairment of the induction of the FN signalling pathway in the MDA5C2 cell line compared to the wild-type (WT) following viral infection. This was the reason for using the MDA5C2 cell line as a control because MDA5C2 was isolated at the same time as MDA5C1 resulting in the cultures being synchronised and at the same passage number. We have added this data in the supplementary file in the revised version.*

- Manuscript needs to be thoroughly edited for grammar, many awkward sentences and atypical word choices (examples include the first sentence of the abstract, the abstract sentence "invalidated for the ifih1 gene", figure 3A y-axis label includes "et" instead of and)

*Figure 3A y-axis was corrected, all other figures were checked. We replaced the term “invalidated” by “knockout”. Grammar was checked by the first author who is a native English speaking scientist (see comment to editor above).*

- If cells were plated and then allowed to grow to confluence, how was MOI determined? Were cells re-counted prior to infection?

*This cell line is well characterised and a monolayer of CHSE-EC that just reached confluency is equivalent to 2.5.10^6^ cells per well of a 6-well plate. We have added this information in the revised version.*

- Was beta-actin check for stability as a housekeeping gene?

*We always check for stability by analysing the distribution of Cq/Ct across the samples. No effect of viral infection could be detected. This gene is widely used in infection studies and we have tested its stability many times. We can cite (among others) the following paper where CHSE cells were infected with an Aquabirnavirus and ISG induction was checked by qPCR (Gamil AAA, Evensen Ø, Mutoloki S (2015) Infection profiles of selected aquabirnavirus isolates in CHSE cells. PLoS One 10:.* [*https://doi.org/10.1371/journal.pone.0134173*](https://doi.org/10.1371/journal.pone.0134173) *).*

- For SHRV and SAV 15ul of RNA was used, how was it confirmed that this RNA came from the same number of cells?

*Concentration of RNA was determined by nanodrop and adjusted to 100ng/µl for all samples. A fixed quantity of RNA (1.5µg) was used for each cDNA reaction. Each reaction corresponds to one well and therefore the same initial number of cells. We have corrected the text in the revised version.*

- NNV details are missing, how was RNA isolated?

*A paragraph was missing – it has been added in the revised version.*

- Clarify in line 186 how FBS was decomplemented

*This was added in the revised version.*

- Describe all abbreviations in figure legends

*This was corrected in the revised version.*

- Discussion should be re-written for flow, several "hanging" paragraphs are present that aren't integrated into the discussion, as an example line 289-291.

*Several paragraphs were moved and rephrased in the discussion to improve the flow.*

While revising your submission, please upload your figure files to the Preflight Analysis and Conversion Engine (PACE) digital diagnostic tool, [https://pacev2.apexcovantage.com/](https://c05y1x9s.r.us-east-2.awstrack.me/L0/https:%2F%2Fpacev2.apexcovantage.com%2F/1/010f019132b94c92-5eb25e65-0979-4fa2-a370-2fb44b586b18-000000/B7SRG51dW_IxeEe2drIMK9Bws48=171). PACE helps ensure that figures meet PLOS requirements. To use PACE, you must first register as a user. Registration is free. Then, login and navigate to the UPLOAD tab, where you will find detailed instructions on how to use the tool. If you encounter any issues or have any questions when using PACE, please email PLOS at [figures@plos.org](mailto:figures@plos.org). Please note that Supporting Information files do not need this step.
